# Power regulation of variable speed multi rotor wind systems using fuzzy cascaded control

**DOI:** 10.1038/s41598-024-67194-4

**Published:** 2024-07-16

**Authors:** Habib Benbouhenni, Ilhami Colak, Nicu Bizon, Mohamed I. Mosaad, Teshome Goa Tella

**Affiliations:** 1 Faculty of Engineering and Architecture, Department of Electrical and Electronics Engineering, Nisantasi University, 34481742 Istanbul, Turkey; 2 Faculty of Engineering and Natural Science, Department of Electrical and Electronics Engineering, Istinye University, Istanbul, Turkey; 3 The National University of Science and Technology POLITEHNICA Bucharest, Pitești University Center, 110040 Pitesti, Romania; 4 ICSI Energy, National Research and Development Institute for Cryogenic and Isotopic Technologies, 240050 Ramnicu Valcea, Romania; 5 Yanbu Industrial College (YIC) Alnahdah, Yanbu Al Sinaiyah, 46452 Yanbu, Saudi Arabia; 6 Sustainable Energy Centre of Excellence, Addis Ababa Science and Technology University, Addis Ababa, Ethiopia

**Keywords:** Cascaded fuzzy power control, Rotor side converter, Multi-rotor wind turbine, Direct power control, Doubly-fed induction generator, Energy science and technology, Engineering

## Abstract

Power quality is a crucial determinant for integrating wind energy into the electrical grid. This integration necessitates compliance with certain standards and levels. This study presents cascadedfuzzy power control (CFPC) for a variable-speed multi-rotor wind turbine (MRWT) system. Fuzzy logic is a type of smart control system already recognized for its robustness, making it highly suited and reliable for generating electrical energy from the wind. Therefore, the CFPC technique is proposed in this work to control the doubly-fed induction generator (DFIG)-based MRWT system. This proposed strategy is applied to the rotor side converter of a DFIG to improve the current/power quality. The proposed control has the advantage of being model-independent, as it relies on empirical knowledge rather than the specific characteristics of the DFIG or turbine. Moreover, the proposed control system is characterized by its simplicity, high performance, robustness, and ease of application. The implementation of CFPC management for 1.5 MW DFIG-MRWT was carried out in MATLAB environment considering a variable wind speed. The obtained results were compared with the direct power control (DPC) technique based on proportional-integral (PI) controllers (DPC-PI), highlighting that the CFPC technique reduced total harmonic distortion by high ratios in the three tests performed (25%, 30.18%, and 47.22%). The proposed CFPC technique reduced the response time of reactive power in all tests by ratios estimated at 83.76%, 65.02%, and 91.42% compared to the DPC-PI strategy. Also, the active power ripples were reduced by satisfactory proportions (37.50%, 32.20%, and 38.46%) compared to the DPC-PI strategy. The steady-state error value of reactive power in the tests was low when using the CFPC technique by 86.60%, 57.33%, and 72.26%, which indicates the effectiveness and efficiency of the proposed CFPC technique in improving the characteristics of the system. Thus this control can be relied upon in the future.

## Introduction

The worldwide energy sector is now experiencing a significant change, driven by the urgent need for power solutions that are both sustainable and robust^1^. Conventional power systems that rely on fossil fuels, which have been the mainstay of energy production, are being subjected to further scrutiny because of environmental concerns and the urgent need to decrease greenhouse gas emissions^1^. As a result, novel hybrid power systems (HPSs) have arisen, effectively combining renewable energy sources (RESs) with conventional fossil-based elements^2,3^. Wind energy (WE) is characterized by plentiful and widespread availability. Wind turbines (WTs) have the distinct benefit of global applicability, as they can be used in both onshore and offshore environments. This sets them apart from other RESs, like as photovoltaic (PV) systems, which are constrained by specific geographic and environmental conditions^3^. WTs are a very accessible and versatile energy choice. Technological advancements have greatly enhanced the cost-effectiveness of WE. The cost of WE has fallen as a result of technological breakthroughs in both WT technology and the power electric components used in the accompanying electrical generators^4^. These WTs can be classified into two main categories: horizontal-axis WTs and vertical-axis WTs. Horizontal axis WTs are the most prevalent kind of WTs globally, both on land and at sea, due to their many benefits over other types. Mostly, a three-bladed WT is used to convert WE into mechanical energy (ME). According to the work done in^5^, the energy gained from the WE is related to the size of the WT and the speed of the WE, as the larger the dimensions of the WT, the greater the energy gained from the WE. Also, the greater the wind speed (WS), the greater the energy gained. The negative of traditional WTs, or what are known as single-rotor WTs (SRWTs), lies in the energy gained from the WE compared to the existing WE. Also, these traditional WTs are affected by the WE that arises between the WTs in the wind farm (WF), which causes a decrease in the WTs efficiency, which negatively affects the resulting electrical energy (EE). Therefore, the researchers proposed using multi-rotor WTs (MRWTs) in stead of traditional SRWTs^6^. This substitution significantly increasing the energy gained from the WE and overcoming the problem of interference between the WTs and the WF. According to the work done in^7^, the energy gained from the WE using MRWT is greater than the energy gained from the WE using traditional WTs. Also, the rotation speed of the MRWT is greater than the rotation speed of the conventional SRWT. These results confirm the importance of using this type of WT in the field of REs, as the use of this type contributes to reducing the size of WFs and reducing the dimensions of the WTs, thus reducing the costs of constructing the WTs. Due to the features of these WTs, they are relied upon in this work to create an energy system, as they are used to convert WE into ME to rotate the generator.

In traditional power systems, which primarily depend on fossil fuels and centralized power production, power quality issues are often linked to the characteristics of the generating and distribution infrastructure. Frequent problems include voltage dips, harmonics, voltage variations, and abrupt disruptions. Moreover, the use of traditional spinning generators might potentially generate harmonics, which can have a negative influence on the overall quality of the electricity being delivered^8^. The complexity of power quality issues increases when using HPSs that are dependent on WE. The inherent unpredictability and intermittent nature of WE resources may lead to fluctuations in power generation, which can subsequently contribute to voltage and frequency instability. The sporadic nature of WE, contingent upon meteorological patterns, may provide challenges in guaranteeing a consistent power supply, particularly during periods of less wind. Furthermore, the integration of power electronics and the variable speed of WTs may generate harmonics and imbalances within the electrical grid^9,10^.

MRWTis characterized by its innovative configuration of multiple rotors on a singular support structure^11^. This configuration offers many advantages in the field of WE generation. Turbines equipped with several rotors have a larger blade area, which enables them to capture a greater amount of WE and generate a higher level of power^12^. MRWTs demonstrate superior performance compared to single-rotor WTs in low WS conditions. If one rotor fails, the remaining rotors will continue functioning, guaranteeing steady power production and improving the dependability of multi-rotor systems^12,13^.

In this paper, WE is used as a primary and natural source for generating EE, and a MRWT is used as an effective and appropriate solution. Numerous generators can be used alongside the WT to generate power. The role of these generators is to convert the ME generated by the WT into EE. Therefore, the generator used must match the capacity of the WT, as a WT and a generator with the same capacity must be used to obtain better results. Asynchronous generators(AGs)^14^, synchronous generators^15^, and direct current generators^16^ are among the most prominent generators that have been used in a WE system to generate EE.

In the WT system, the doubly-fed induction generator (DFIG) is considered the most widely used generator, especially in variable WSs^17^. This is mostly owing to its several notable advantages, including its cost-effectiveness and minimal maintenance requirements in comparison to other generator types^18^. DFIG is considered one of the types of AGs that have distinguished performance and high operational efficiency compared to many types, making it one of the most important solutions that can be relied upon in the field of RESs. The use of this generator along with the MRWT is considered the appropriate solution in this paper to generate EE and overcome the defects found in energy systems that rely on traditional WTs. The most major challenges facing any energy system are cost, complexity, ease of realization, outstanding performance, EE quality, durability, life span, and dynamic response. All of these mentioned challenges are necessary and must be dealt with, as these challenges are related to the control strategy. The latter is largely responsible for the performance, system complexity, robustness, and EE quality. Therefore, it is necessary to choose the appropriate command strategy to control the power system to achieve minimal current fluctuations and ensure optimal performance.

In the field of command, many strategies have been designed to command electrical machines, especially generators, where linear controls are famous for their simplicity and fast dynamic response^19^. The direct power command (DPC) strategy is considered one of the most famous linear strategies and the easiest to achieve, as it relies on the use of a hysteresis comparator (HC) to regulate the DFIG power and on estimation to calculate the power error^20^. Also, a switching table (ST) is used to generate the necessary pulses to control the rotor side converter (RSC) of DFIG, which makes unwanted frequencies present at the inverter output. The DPC has a very fast dynamic response, which makes it most suitable in the area of command. Also, this strategy is characterized by ease of application to complex systems, low charge, few gains, and ease of implementation compared to many other controls. This strategy has disadvantages such as power ripples(active and reactive power (*Ps* and *Qs*)), high value of total harmonic distortion (THD) of current, and low robustness when a malfunction occurs in the DFIG^21^. These difficulties are undesired and have a negative impact on both the network and the power system itself. Due to the importance of the DPC strategy, several solutions were proposed in the field of control to overcome its shortcomings. The most famous of these solutions relied on changing traditional controllers to increase performance. In Table 1, the proposed solutions are collected to improve the characteristics of the strategy, where the proposed controller to increase the characteristics of the DPC strategy of DFIG is mentioned, along with the negatives and positives.

**Table 1:** Some proposed solutions to overcome the problems of the DPC strategy of DFIG.

References	Type of study	The type of controller used to improve DPC performance	Type of turbine	Cons	Positives
^22^	Simulation	New look-up table	Traditional turbine	Use energy estimation, Susceptibility to changing system parameters, energy ripples, and high THD of current	Simplicity, ease of implementation, fast dynamic response, and few gains
^23^	Simulation	Synergetic sliding mode controller (SMC)	MRWT	Complexity, capacity estimation, cost, number of gains, difficulty of completion, and response time	Reducing power surges, improving current quality, increasing robustness, and outstanding performance
^24^	Simulation	Fuzzy SMC technique	Traditional turbine	The number of rules of fuzzy logic (FL), the use of a mathematical model (MM)of the system, the number of gains, complexity, estimation of capabilities, difficulty of completion, and response time	High robustness, overcome DPC strategy problems, lower THD value
^25^	Simulation	Fractional-order proportional integral super-twisting SMC technique	Traditional turbine	Complexity, cost, energy estimation, difficulty of completion, number of gains, and response time	Overcome DPC strategy problems, robustness, and improving current quality
^26^	Simulation	Backstepping control (BC)	Traditional turbine	Complexity, estimation of energies, relying on the MM of the system, being affected by changing system parameters, difficulty of completion, and response time	Robustness, reduce power ripples, minimize the THD value of current
^27^	Experimental	Artificial neural network (ANN)	Traditional turbine	The number of internal layers needed to obtain good results, power estimation, and number of cells in each layer	Ease of implementation, fast dynamic response, does not depend on the MM of the system, a small number of gains, is not affected by internal and external factors of the system, reduces power ripples, minimizes the THD value of current
^28^	Experimental	Super-twisting control (STC)	Traditional turbine	Number of gains, estimation of capabilities, and response time	Reduce power ripples, and minimize the THD value of current, simplicity, and robustness
^29^	Simulation	Proportional-integral (PI) controller based on genetic algorithm (GA) and terminal sliding surface technique	MRWT	Estimating capabilities, number of gains, expensive, difficult to achieve, number of gains, and response time	Overcome the problems of DPC strategy, greatly increasing robustness and performance
^30^	Simulation	FL technique	Traditional turbine	The number of rules of FL, the number of gains, estimation of energies, and response time	High robustness, overcome DPC strategy problems, lower THD value
^31^	Simulation	SMC technique	Traditional turbine	Reliance on the MM, the phenomenon of chattering, complexity, the difficulty of completion, and the use of energy estimation	Reducing ripples, increasing robustness, improving performance and efficiency, and improving current quality
^32^	Simulation	Modified SMC technique	MRWT	Power estimation	Simplicity, ease of realization, high robustness, outstanding performance, small number of gains, fast dynamic response, and reduced overshoot
^33^	Simulation	Simplified STC technique	MRWT		
^34^	Experimental	Feedback PI controller	MRWT		
^35^	Simulation	Dual STC technique	MRWT	Power estimation, complexity, expensive, difficult to achieve, and large number of gains	Improving the values ​​of overshoot and steady-state error (SSE), increasing the quality of current and power, improving robustness and performance, reducing the value of THD of current, and overcoming the problems of the DPC strategy
^36^	Simulation	Neural STC technique	MRWT	Determining the number of internal layers needed, the number of neurons in each layer, estimating energies, affected by changing system parameters, and response time Time to set gain values	
^37^	Experimental	Intelligent STC technique	MRWT		
^38^	Simulation	GA-based STC technique	–		
^39^	Simulation	Integral BC technique	Traditional turbine	Complexity, number of gains, use of the MM of the DFIG, difficulty of completion, and estimation of energies	Reducing the value of THD of current, and overcoming the problems of the DPC strategy Improving the values of overshoot and SSE, increasing the energy/current quality, improving robustness and performance
^40^	Simulation	Synergetic controller	Traditional turbine	The presence of ripples at the energy and current levels, affected by changing the parameters, the THD value of current, and power estimation	
^41^	Simulation	Neural PI controller	Traditional turbine	Choosing the type of neural network and learning algorithm, dynamic response, affected by changing parameters, power estimation, THD value of current, and reduced robustness in case of changing parameters	
^42^	Simulation	Fractional-order PI controller	Traditional turbine	The number of gains is affected by changing system parameters, the presence of ripples at the level of both power and current, and the estimation of powers	
^43^	Simulation	BC technique with nonsingular terminal sliding mode surface technique	MRWT	Complexity, a large number of gains, response time, power estimation, affected by changing parameters, dependence on the MM of the DFIG, and power estimation	
^44^	Simulation	Fractional-order neural controller	MRWT	Choosing the type of ANN and learning algorithm, and power estimation	Improving performance and robustness, increasing power and current quality, and improving the values of both SSE and overshoot Overcoming DPC strategy problems
^45^	Simulation	GA-based type-I FL controller	Traditional turbine	The number of FL rules, the number of gains, and the time for calculating parametric values, and power estimation	
^46^	Simulation	Sliding-backstepping mode control	Traditional turbine	Complexity, its dependence on the MM of the DFIG, difficulty of application, the problem of chatter, low quality of current and power in the robustness test, capacity estimation, and response time	
^47^	Simulation	Intelligent modified SMC technique	MRWT	Low current quality in robustness test and power estimation	
^48^	Simulation	Synergetic-PI control based on GA technique	MRWT	Complexity, large number of gains, estimation of powers, response time, and difficulty of completion	
^49^	Simulation	Super-twisting fractional-order terminal SMC technique	Traditional turbine		
^50^	Simulation	Thirde-order SMC technique	MRWT		

Traditionally, the FL strategy is considered one of the most important strategies that can be relied upon in the field of controlling electrical machines because of its many and varied features, as this strategy is considered one of the strategies that do not depend on the MM of the system under study^51^. Also, it is characterized by high robustness against internal and external factors of the system, which allows its use to obtain very satisfactory results. This strategy relies heavily on experience and the use of rules called FL rules, which differ from traditional logic. Due to its ability to greatly improve the characteristics of systems, it has been relied upon in several different fields, where it has been used to control the asynchronous machine^52^, the photovoltaic system^53^, the dual star induction generator^54^, and the DFIG power^55^. In the work^56^, the FL strategy with fractional calculus was used to control the powers of the DFIG-MRWT controlled by the DPC strategy. A fractional-order FL control was used to overcome the problem of power ripples and reduced robustness while changing the DFIG parameters, given that the outputs of these controls serve as reference voltage values. These values are converted using the PWM strategy into pulses to operate the DFIG inverter, where power estimation is used to determine the power error. The latter is required to calculate reference voltage values. Therefore, this proposed strategy is characterized by simplicity, ease of implementation, outstanding performance, and high robustness. Also, it is characterized by a fast dynamic response. 49 rules were used to implement the proposed controller, as this number of rules allows for excellent results. This designed technique was implemented in the MATLAB (https://www.mathworks.com/products/new_products/release2022a.html) using several different tests with a comparative study with other controls in terms of ripple reduction ratios, overshoot, and SSE of DFIG power. All completed test results show the high performance of the DPC strategy based on fractional-order FL control in terms of the value of current THD, ripples, overshoot, and SSE compared to the DPC strategy.In the work^57^, the author combined the FL strategy with a NN to overcome the problems and drawbacks of the DPC of the DFIG strategy. This proposed solution provided satisfactory results, as shown by the results of the MATLAB (https://www.mathworks.com/products/new_products/release2022a.html) under different working conditions for DFIG. Therefore, relying on the FL technique as a suitable solution gives satisfactory results. As is known, robustness and performance are two of the most important features that must be paid attention to for any control strategy. Also, to obtain high-quality power and current, a controller with high specifications must be chosen. All of the solutions proposed above depend on modifying the DPC strategy to control energies, using other strategies, which in some cases increases the degree of complexity and difficulty of implementation. Therefore, it is necessary to search for a more suitable control strategy to control the DFIG power.

In this paper, a new cascaded fuzzy power control (CFPC) is proposed to control DFIG-MRWT energy. The idea is based on using the cascaded FL technique in place of two HCs to control the powers, and the MPPT technique is used to commandthe RSC of DFIG-MRWT. In this way, the simplicity, low cost, ease of application, and quick dynamic response that characterize the DPC are maintained. In addition to greatly increasing robustness as a result of the use of the CFPC technique, which is characterized by robustness and is not affected by changes in system parameters.

CFPC technique using the PWM technique is a new strategy that has not been dealt with in this previously proposed structure in any work, as it differs from the DPC strategy and several other papers^38,46,49,50^. So, the CFPC-PWM technique is the main contribution of this paper, as the tests performed have highlighted its ability to minimize energy ripples and significantly increase the current quality. The CFPC-PWM technique was applied to the RSC only to demonstrate the extent of its ability to reduce energy fluctuations and improve the characteristics of the proposed energy system. The MATLAB, Matlab/simulink 2022 (https://www.mathworks.com/products/new_products/release2022a.html) was used for verifying the validity, effectiveness, and ability of this strategy compared to the DPC strategy. Also, a comparison with existing papers was achieved in terms of minimizing the ratios of response time, energy ripple, SSE, and overshoot. In this work, a variable WS was used to study the effectiveness and robustness of the CFPC-PWM technique using a 1500 kW DFIG-MRWT system.

Several objectives have been achieved through this work, which can be explained in the following points:

- Reduction of energy ripples compared to the DPC-PI technique and other strategies recently proposed in the specialized literature;
- Reducing the THD value of the current compared to several scientific works;
- Significantly increasing the system robustness;
- Reducing the values of both overshoot and SSE of DFIG power compared to several works.
- Developing a new strategy different from several existing strategies.

The paper is based on the following sections: The second section deals with the energy system proposed for the study, where the MM for both MRWT and DFIG is given, mentioning the negatives and positives of the proposed system. In addition, to gives a simulation of the MRWT, which demonstrates its effectiveness and distinctive performance compared to traditional turbines. In the third section, numerical and graphical results for both DPC and CFPC-PWM techniques are given, along with a comparison with other scientific papers. Finally, the paper ends with a section in which all obtained results are summarized.

## Designed energy system

**Figure 1:**
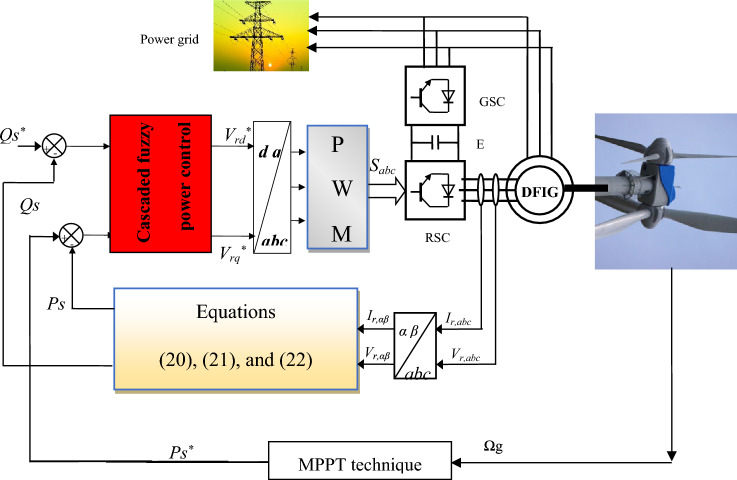
Fuzzy cascaded power control of DFIG-MRWT system.

The energy system designed for this study is represented in Fig. 1, as it is characterized by simplicity, lowcharge, uncomplicated, and easy control. This system contains MRWT, DFIG, RSC, and GSC. In addition to the control strategy, this proposed system contributes significantly to significantly reducing the production bill and energy consumption while preserving the environment.

*RSC control:* The PWM was used for command because of its simplicity and ease of application, which makes the designed system less complex and, therefore less expensive and easy to maintain.

*GSC control:* To simplify the system and show the extent of the impact of the designed command in reducing EE ripples, a GSC was proposed using a diode, as no control is used for it. In this way, it is possible to know the performance and efficiency of the CFPC-PWM technique compared to the DPC technique and some other papers in minimizing the value of current THD and the robustness of the system.

*Power control:* The CFPC-PWM technique is used as a suitable solution for power control because of its robustness and lack of relation to the MM system.

*Energy estimation:* This process is necessary forthis designed system since it requires knowledge of the error in the powers to calculate the voltage reference values. Although the estimation of power is related to the resistance (*Rs*), this process has been relied upon because it is necessary to extract the control pulses in the RSC.

*Turbine control:* The maximum power point tracking (MPPT) strategy based on the PI controller is used to control the MRWT and extract maximum power from the WE. This strategy was relied upon to simplify control and not complicate it.

The MPPT-PI strategy used in this work is the same strategy detailed in the work^7^, where the same characteristics of the MRWT were used. Using the MPPT-PI strategy to determine the reference value for *Ps* makes the current, torque, and *Ps* related to the nature of the WS.

### DFIG model

The power in this proposed system is produced using DFIG. Due to the many advantages compared to other generators, it is necessary to complete a MM to be used in simulation. The Park transform is used for this purpose to extract the equations needed for DFIG^45,46^. Equation (1) represents both the flux and voltage of the rotor of the DFIG^50^.

1 $$ \left\{ {\begin{array}{*{20}c}    {V_{{dr}}  = R_{r} I_{{dr}}  + \frac{d}{{dt}}\Psi _{{dr}}  - w_{r} \Psi _{{qr}} }  \\    {\Psi _{{dr}}  = L_{r} I_{{dr}}  + MI_{{ds}} }  \\    {V_{{qr}}  = R_{r} I_{{qr}}  + \frac{d}{{dt}}\Psi _{{qr}}  + w_{r} \Psi _{{dr}} }  \\    {\Psi _{{qr}}  = L_{r} I_{{qr}}  + MI_{{qs}} }  \\   \end{array} } \right. $$

where, *Ѱ*_*dr*_ and *Ѱ*_*qr*_ are the rotor fluxes, *I*_*dr*_ and *I*_*qr*_ are the rotor currents, *M* is the mutual inductance, *V*_*dr*_ and *V*_*qr*_ are the rotor voltages, *L*_*r*_ is the inductance of the rotor, and *R*_*r*_ is the rotor resistance.

In Eq. (2), the relationship between flux, voltage and DFIG stator current is shown. The fixed part is the one that is connected directly to the network without an intermediary^19^.

2 $$\left\{\begin{array}{c}{V}_{ds}={R}_{s}{I}_{ds}+\frac{d}{dt}{\Psi }_{sd}-{w}_{s}{\Psi }_{qs}\\ {\Psi }_{ds}={L}_{s}{I}_{ds}+M{I}_{dr}\\ {V}_{qs}={R}_{s}{I}_{qs}+\frac{d}{dt}{\Psi }_{qs}+{w}_{s}{\Psi }_{ds}\\ {\Psi }_{qs}={L}_{s}{I}_{qs}+M{I}_{qr}\end{array}\right.$$

where, *Ѱ*_*ds*_ and *Ѱ*_*qs*_ are the stator fluxes, *ω*_*s*_ is the electrical pulsation of the stator, *L*_*s*_ is the inductance of the stator, *V*_*ds*_ and *V*_*qs*_ are the stator voltages, and *R*_*s*_ is the stator resistance.

The relationship between the speed and torque of the DFIG is shown in Eq. (3), as this equation shows the development of speed as a function of torque. This development turns the machine into a generator or engine state. In addition, the equation explains the torque expression used in this work.

3 $$\left\{\begin{array}{c}{T}_{e}-{T}_{r}=J\frac{d\Omega }{dt}+f\Omega \\ {T}_{e}=\frac{3}{2}p\frac{M}{{L}_{s}}({\Psi }_{sq}{I}_{rd}-{\Psi }_{sd}{I}_{rq})\end{array}\right.$$

where, *T*_*e*_ is the torque, *J* is the inertia, *p* is the number of pole pairs, *Ω* is the mechanical rotor speed, *f* is the viscous friction coefficient, and *T*_*r*_ is the load torque.

The DFIG energy is represented in Eq. (4), where power is closely related to voltage and current, and the quality of the energy is related to the current quality.

4 $$\left\{\begin{array}{c}{P}_{s}=\frac{3}{2}(+{V}_{qs}{I}_{qs}+{V}_{ds}{I}_{ds})\\ {Q}_{s}=\frac{3}{2}(+{V}_{qs}{I}_{ds}{-V}_{ds}{I}_{qs})\end{array}\right.$$

By making the flux a constant value and directing it along the d-axis and neglecting the resistance (*R*_*s*_), the flux can be written according to Eq. (5). This equation is used to simplify the MM of the DFIG and control itself.

5 $${\Psi }_{ds}=0 \text{and}{ \Psi }_{qs}={\Psi }_{s}$$

With:

6 $$\left\{\begin{array}{c}{V}_{qs}={V}_{s}={w}_{s}\cdot {\Psi }_{s}\\ {V}_{ds}=0\end{array}\right.$$

By applying the Eqs. (5) and (6), the stator currents can be written according to Eq. (7).

7 $$\left\{\begin{array}{c}{I}_{qs}=-\frac{M}{{L}_{s}}{I}_{qr}\\ {I}_{ds}=-\frac{M}{{L}_{s}}{I}_{dr}+\frac{{\Psi }_{s}}{{L}_{s}}\end{array}\right.$$

The Eq. (4) can be written in the following form:

8 $$\left\{\begin{array}{c}{Q}_{s}=-\left(\frac{{V}_{s}M}{{L}_{s}}{I}_{dr}+\frac{M.{{V}_{s}}^{2}}{{{\Psi }_{s}L}_{s}}\right)\\ {P}_{s}=-\frac{{V}_{s}M}{{L}_{s}}{I}_{qr}\end{array}\right.$$

The torque expression in Eq. (3) becomes the form represented by Eq. (9).

9 $${T}_{e}=-{I}_{qr}{. \Psi }_{s}. p.\frac{M}{{L}_{s}}$$

The rotor currents of the DFIG can be written according to Eq. (10).

10 $$\left\{\begin{array}{c}{I}_{qr}=\left({V}_{qr}-g\cdot {w}_{s}\left({L}_{r}-\frac{{M}^{2}}{{L}_{s}}\right){I}_{qr}-g\frac{M\cdot {V}_{s}}{{L}_{s}}\right)\frac{1}{{R}_{r}+\left({L}_{r}-\frac{{M}^{2}}{{L}_{s}}\right)p}\\ {I}_{dr}=\left({(V}_{dr}-g\cdot {w}_{s}({L}_{r}-\frac{{M}^{2}}{{L}_{s}}){I}_{qr}\right)\frac{1}{{R}_{r}+\left({L}_{r}-\frac{{M}^{2}}{{L}_{s}}\right)p}\end{array}\right.$$

Hence, *Vqr* and *Vdr* can be written as:

11 $$\left\{\begin{array}{c}{V}_{qr}={R}_{dr}\cdot {I}_{qr}-g\cdot {w}_{s}\left({L}_{r}-\frac{{M}^{2}}{{L}_{s}}\right){I}_{dr}+g\frac{M\cdot {V}_{s}}{{L}_{s}}\\ {V}_{dr}={R}_{dr}\cdot {I}_{dr}-g\cdot {w}_{s}({L}_{r}-\frac{{M}^{2}}{{L}_{s}}){I}_{qr}\end{array}\right.$$

where, $$g = \frac{{\omega_{r} }}{{\omega_{s} }}$$.

### MRWT model

Turbines are in continuous and permanent development because of their great importance in the Resfield, as they have received great interest from researchers and manufacturers. Recently, it has been noted that the number of WFs on land and sea has increased to generate EE, as their use contributes significantly to protecting the environment and reducing toxic gas emissions. Additionally, the use of WTs plays a substantial role in diminishing the expenses associated with generating RE, which is a favourable outcome. MRWT is a new WT that has emerged as a suitable solution to leverage the power gained from WE and overcome the problems of traditional WTs.This technology is detailed in^12,45,51^, where several WTs of different sizes can be used to form a single turbine. To control this WT, the MPPT strategy is used for this purpose, as the MPPT of MRWT is considered complex and difficult to implement compared to the MPPT strategy of traditional WTs. This technology is in continuous development despite its novelty and has shown outstanding performance in increasing the power gained from the WE. The energy gained from WE can be expressed by Eq. (12), where this power is the sum of the energy of each WT^42^.

12 $${T}_{MRWT}=\sum_{1}^{n=2}{T}_{n}$$

where, the *T*_*MRWT*_ is the torque of the mother WT and *T*_*n*_ is the torque for the WTmother with *n* = 1 and 2.

The torque for each WT is represented in Eq. (13), where this torque is related to the dimensions of each WT and the WS^43^.

13 $$\left\{\begin{array}{c}{T}_{2}=\frac{{C}_{p2}}{2{\lambda }_{2}^{3}}\cdot {R}_{2}^{5}\cdot {w}_{2}^{2}.\rho \cdot \pi \\ {T}_{1}=\frac{{C}_{p1}}{2{\lambda }_{1}^{3}}\cdot {R}_{1}^{5}\cdot {w}_{1}^{2}.\rho \cdot \pi \end{array}\right.$$

where, *λ*_*1*_ and *λ*_*2*_ are the tip speed ratio of the both rotors, *ρ* is the air density, *w*_*2*_ and *w*_*1*_ are the mechanical speed of both rotors, and *R*_*2*_ and *R*_*1*_ are the blade radius of both rotors.

Equation (14) expresses the power gained from the WE as it relates to the WT power. This resulting energy is related to a parameter called the coefficient of power (*Cp*), which is expressed by Eq. (15).

14 $${P}_{MRWT}=\sum_{1}^{n=2}{P}_{n}$$

where, the *P*_*MRWT*_ is the energy of the mother WT and *P*_*n*_ is the power for the WT mother with *n* = 1 and 2.

15 $${C}_{p}\left(\lambda ,\beta \right)=-\frac{0.035}{{\beta }^{3}+1 }+\frac{1}{\lambda +0.08\cdot \beta }$$

where, *β* is the pitch angle.

Equation (16) represents the tip speed ratios of the both WTs^47^.

16 $$\left\{\begin{array}{c}{\lambda }_{2}=\frac{{w}_{2}\cdot {R}_{2}}{{V}_{2}}\\ {\lambda }_{1}=\frac{{w}_{1}\cdot {R}_{1}}{{V}_{1}}\end{array}\right.$$

where,*V*_*1*_ and *V*_*2*_ are the tip speed ratios of the rotor, where *V*_*1*_ ≠ *V*_*2*_.

*V*_*1*_ is the WS of the first WT and is equal to the WS*V* (*V*_*1*_ = *V*). But the WS of the second WT differs from V, so depending on the work^29^ it can be calculated according to Eq. (17). This speed is related to the distance between the two WTs (*x*), which is estimated at 15 m, and to the WS of the first WT (*V*_*1*_).

17 $${V}_{2}={V}_{1}\cdot \left(1-\frac{1-\sqrt{\left(1-{C}_{T}\right)}}{2}\left(1+\frac{2x}{\sqrt{1+4{x}^{2}}}\right)\right)$$

where, *C*_*T*_ is the trust coefficient (*C*_*T*_ = 0.9)^45,49^.

**Figure 2:**
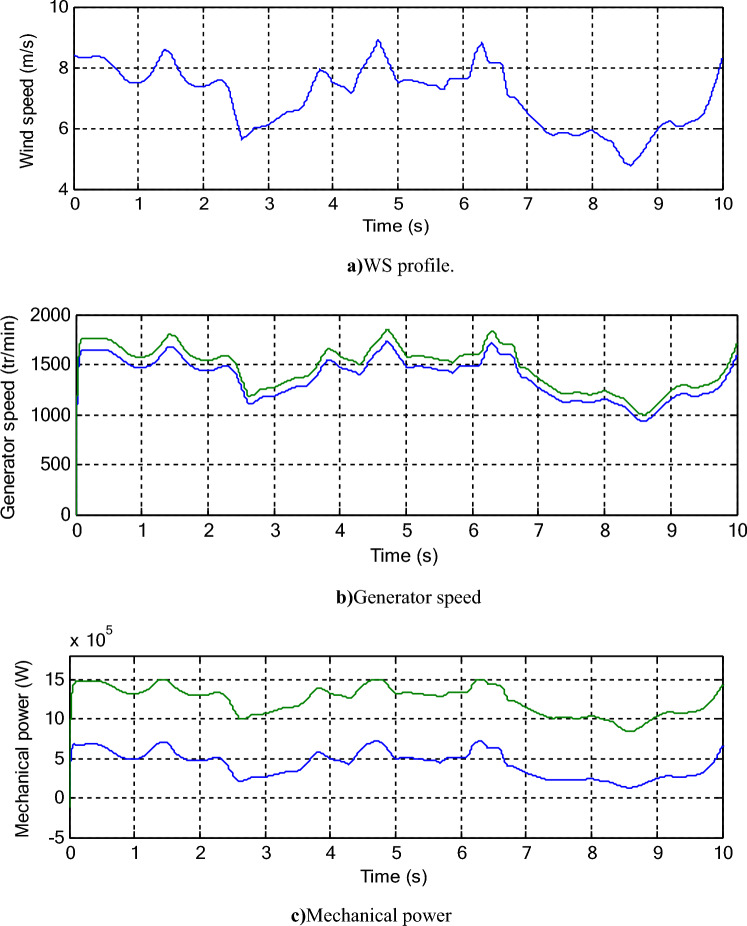
Turbine simulation results.

In Fig. 2, the simulation results are given for a regular WT(single rotor WT (SRWT) an MRWT, where the two WTs have the same power 1500 kW). Numerical results are represented in Tables 2 and 3. The used MRWT parameters are as follows: Numberof turbine blades = 3, *R*_*2*_ = 25.5 m, number of secondary WT blades = 3, *r*_*2*_ = 0.5 m, *r*_*g*_ = 0.75 m, *R*_*1*_ = 13.2 m, *r*_*1*_ = 1 m, *J*_*2*_ = 1000 kg m^2^, *J*_*1*_ = 500 kg m^2^^49,51^.

**Table 2:** Speed value of both WTs.

Times (s)	Generator speed (rot/min)		Ratios (%)	Average ratio (%)
	SRWT	MRWT		
1	1471	1574.70	6.58	6.63
2	1441.50	1543.65	6.61	
3	1185.20	1269.45	6.63	
4	1478.18	1583.17	6.63	
5	1480	1585.55	6.65	
6	1488.83	1594.75	6.64	
7	1274.28	1365.15	6.65	
8	1154.63	1236.93	6.65	
9	1146.73	1228.35	6.64	
10	1588.52	1701.32	6.63

**Table 3:** Mechanical power of both WTs.

Times (s)	Mechanical power (MW)		Ratios (%)	Average ratio(%)
	SRWT	MRWT		
1	0.49	1.31	62.59	63.14
2	0.647	1.29	49.84	
3	0.62	1.06	51.50	
4	0.49	1.32	62.87	
5	0.49	1.32	62.87	
6	0.51	1.33	61.65	
7	0.31	1.14	72.80	
8	0.24	1.03	76.69	
9	0.24	1.02	76.47	
10	0.65	1.42	54.22	

The WS of the Moroccan city of Dakhla is used for a comparative study between the two WTs, where the shape of the change in this speed is represented in Fig. 2a. The rotation speed of the DFIG used with the two WTs is represented in Fig. 2b, where the rotation speed of the DFIG changes according to the change in WS. This speed increases and decreases with the increase and decrease in WS. Also, the rotational speed of the DFIG in the case of MRWT is greater than the rotational speed of the DFIG in the case of a conventional WT. From Fig. 2b, the smallest value of the rotational speed of a DFIG in the case of a conventional WT was 8.60 s at the moment. Its value reached 931.33 rot/min, and in the case of the MRWT, the lowest speed of the DFIG was 997.80 rot/min at the moment of 8.60 s. Therefore, the MRWT provided a greater value for the lowest DFIG speed than the traditional SRWT. The largest DFIG speed value at the moment was 4.71 s for the two types, where the speed value reached 1732 rot/min and 1855.13 rot/min for both SRWT and MRWT, respectively. Therefore, MRWT provided the largest value compared to SRWT, with an estimated ratio of 6.63%. This percentage proves the superiority of the MRWT and its ability to improve the characteristics of the WE system. In Table 2, the speed values at different moments are given when using both SRWT and MRWT, where improvement percentages are calculated from the speed value during each time point. From Table 2, it is noted that the MRWT provided a rotation speed greater than the rotation speed provided by the ordinary WT during the various given moments. Accordingly, from the table, it is noted that the MRWT provided rotation speed improvement ratios ranging from 6.58% to 6.65% compared to the conventional WT. So, an MRWT can improve the rotational speed of the WT by an average of 6.63% compared to a regular WT, providing the distinctive performance of this WT and its effectiveness.

The energy gained from the WE for the two WTs is represented in Fig. 2c, where the value of this gained energy changes according to the change in WS. Also, the energy gained from WE is greater in the case of using an MRWT compared to a regular WT. From Fig. 2c, the largest value of energy gained from the WE for a conventional WT is estimated at about 0.72 MW in 4.71 s, and for an MRWT, the largest value of energy was 1500 kW at the same moment in time. The lowest value of the energy gained from the WE was at the moment of 8.60 s for the two WTs, where it was estimated at 0.126 MW and 0.831 MW for both SRWT and MRWT, respectively. The MRWT provided the greatest energy gain from the WE during different periods, where these periods are 1.4 s to 1.48 s, 4.64 s to 4.79 s, and 6.24 s to 6.36 s, which indicates that this WT has the ability to generate greater energy from the WE during different periods. In Table 3, the values of the energies gained from the WE are given if the two WTs were used during different periods. It is noted that the MRWT during these periods provided more energy than the SRWT, as the MRWT provided greater energy with an average rate of 63.14% compared to the SRWT.

### Proposed strategy

The strategy proposed in this work differs from the DPC-PI technique in terms of robustness, performance, simplicity, and efficiency in reducing undulations. Therefore, before discussing the designed technique, we must first give an overview of the DPC-PI of the DFIG because the designed technique is a modification of this strategy.

### A. DPC-PI technique

Traditionally, the DPC-PI strategy is considered one of the most famous solutions proposed to overcome the problems of the DPC, as it relies on using of a PI controller to regulate the distinct amounts. In the DPC-PI strategy, the SVM or PWM technique is used to convert the voltage reference values generated by the PI controller into the pulses needed to operate the DFIG inverter. The DPC-PI strategy is characterized by simplicity, ease of implementation, inexpensiveness, and rapid dynamic response. Also, this strategy uses a PI controller, which makes it one of the most prominent strategies that contains a small number of gains and is, therefore, easy to adjust and change the dynamic response to energies.

**Figure 3:**
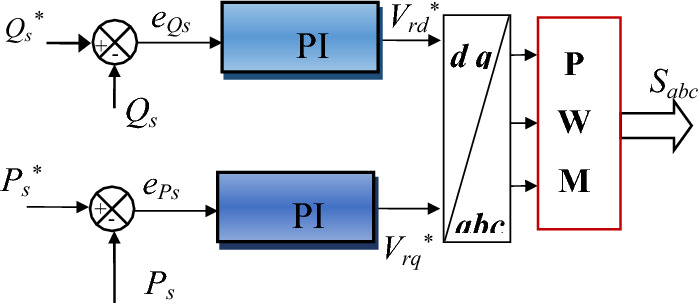
Block diagram of the internal structure of the DPC-PI technique for RSC.

A Fig. 3 represents the principle of the DPC-PI strategy of DFIG, where the PWM strategy was used to generate the pulses necessary to operate the DFIG inverter. In this strategy, the power error is calculated to determine the reference values for the voltage, as these reference values are the outputs of the PI controllers. Therefore, to determine the power error, it is necessary first to estimate the power.

Equation (18) represents the errors in the energies used in the DPC-PI technique. To calculate these errors, the energies must first be estimated, and the voltage and current are measured for this purpose.

18 $$\left\{\begin{array}{c}{e}_{Ps}=-{P}_{s}+{P}_{s}^{*}\\ {e}_{Qs}=-{Q}_{s}+{Q}_{s}^{*}\end{array}\right.$$

The reference value of *Ps* is calculated according to the MPPT, where the value of *Ps* becomes largely related to the changes in the WS profile.

In the DPC-PI technique, the equations for estimating energies are the same as those used in the DPC. Estimating energies is linked to first estimating each of the rotor and stator fluxes. Equation (19) can be used to calculate the stator flux in the stator of the DFIG^21,47^.

19 $$\left\{\begin{array}{c}{\Psi }_{s\beta }=\sigma {I}_{r\beta }{L}_{r}\\ {\Psi }_{s\alpha }=\sigma {I}_{r\alpha }{L}_{r}+{\Psi }_{s}\frac{M}{{L}_{s}}\end{array}\right.$$

where, $$\sigma =1-\frac{{M}^{2}}{{L}_{s}{L}_{r}}$$

Equation (20) can be used to estimate the flux in the moving part of the DFIG, as measuring voltage and current is necessary for this purpose.

20 $$\left\{\genfrac{}{}{0pt}{}{{\Psi }_{r\beta }={\int }_{0}^{t}({V}_{r}-{R}_{r}\times {i}_{r\beta })dt}{{\Psi }_{r\alpha }={\int }_{0}^{t}({V}_{r}-{R}_{r}\times {i}_{r\alpha })dt}\right.$$

Using Eqs. (19) and (20), the flux values are calculated according to the Eq. (21).

21 $$\left\{\begin{array}{c}\left|{\Psi }_{r}\right|=\sqrt{\left({\Psi }_{r\beta }^{2}+{\Psi }_{r\alpha }^{2}\right)}\\ \left|{\Psi }_{s}\right|=\sqrt{\left({\Psi }_{s\beta }^{2}+{\Psi }_{s\alpha }^{2}\right)}\end{array}\right.$$

Using the previous equations, the energies are estimated according to Eq. (22)^20,50^.

22 $$\left\{\begin{array}{c}{Q}_{s}=-\frac{3}{2}\left(\frac{{V}_{s}}{{\sigma \times L}_{s}}{\times \Psi }_{\beta r}-\frac{{V}_{s}{\times L}_{m}}{{\sigma {\times L}_{r}\times L}_{s}}\right)\\ {P}_{s}=-\frac{3}{2}{V}_{s}{\times \Psi }_{r\beta }\times \frac{{L}_{m}}{{\sigma \times {L}_{r}\times L}_{s}}\end{array}\right.$$

The DPC-PI technique's reliance on a PI-type controller to control power makes this strategy less robust, which is a negative. According to the work done in^59^, the DPC-PI technique is affected by the change in DFIG parameters, as an increase in the value of the power ripples and the value of the current THD is observed, which is an undesirable matter that makes it necessary to search for the best continuous control. Therefore, to overcome the problems and drawbacks of the DPC-PI technique and the DPC strategy of DFIG, the solution lies in the strategy proposed in the next subsection.

### B. CFPC-PWM technique

**Figure 4:**
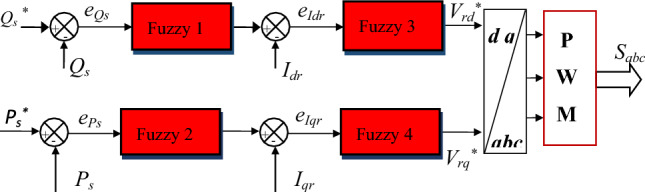
Block diagram of the internal structure of the CFPC strategy.

In this section, a new technique is designed based on using the FL technique, where both the four FL controllers and the PWM strategy are used for this purpose. So the CFPC-PWM technique is the new control that was relied upon in this paper to overcome the problems of both DPC and DPC-PI, as it is considered a new strategy that relies on the use of the FL strategy because of its robustness, not being affected by the internal and external factors of the system, and does not use the MM of the system. This strategy is an innovative development of the DPC-PI strategy, where energy estimation is relied upon and the same equations found in both DPC and DPC-PI are used. Accordingly, Fig. 4 gives a clear picture of the principle of the designed technique for controlling the DFIG inverter, as this strategy is applied to the DFIG inverter only without the network inverter to simplify the system and reduce its total costs.

Also, to demonstrate the ability of the proposed CFPC-PWM strategy to improve the quality of power and current without resorting to controlling the network inverter.

In this proposed CFPC-PWM strategy, the reference values of the rotor current (*I*_dr_^*^ and *I*_qr_^*^) are first calculated based on the error powers (*e*_*Ps*_ and *e*_*Q*s_). To calculate the reference values of the currents, Fuzzy 1 and Fuzzy 2 are used for this purpose, where Eq. (23) is used to calculate these reference values.

23 $$\left\{\begin{array}{c}{I}_{qr}^{*}=(Fuzzy \,2 \left({e}_{Ps}\right))\\ {I}_{dr}^{*}=(Fuzzy \,1 \left({e}_{Qs}\right))\end{array}\right.$$

where, *e*_*Ps*_ and *e*_*Q*s_ are the surface of the DFIG power.

The MPPT strategy is used to determine the reference value for *Ps*. Using this strategy makes the reference value for *Ps* change according to the change in WS, and the same goes for the measured value of *Ps*. Also, using the MPPT strategy makes the torque and current change according to the change in the shape of the WS, which is a positive thing that allows obtaining maximum values.

In the CFPC-PWM technique, Fuzzy 3 and Fuzzy 4 are used to calculate the voltage reference values(*V*_dr_^*^ and *V*_qr_^*^) according to Eq. (24). It is noted that the calculation of these reference values is not linked to the system parameters, which makes the CFPC-PWM technique more robust.

24 $$\left\{\begin{array}{c}{V}_{qr}^{*}=(Fuzzy\, 4\left({e}_{Iqr}\right))\\ {V}_{dr}^{*}=(Fuzzy\, 3 \left({e}_{Idr}\right))\end{array}\right.$$

where, ${e}_{Idr}$ and ${e}_{Iqr}$ are the surface or error of the DFIG rotor current.

In the proposed CFPC-PWM strategy, the reference values of the voltage are calculated based on the line values of the currents, which makes this strategy completely different from other strategies, especially the DPC-PI of the DFIG strategy. Therefore, this designed CFPC-PWM strategy is considered a new strategy and has not been discussed before, as it does not use the parameters of the system under study, which makes it give excellent results in the event of a fault in the system. From a first look at this proposed CFPC-PWM strategy, it can be said that the number of gains is negative for this strategy, as there is a significant number of gains as a result of using four FL-type controllers, and in each controller there are 3 gains, making their total 12 gains.

**Figure 5:**
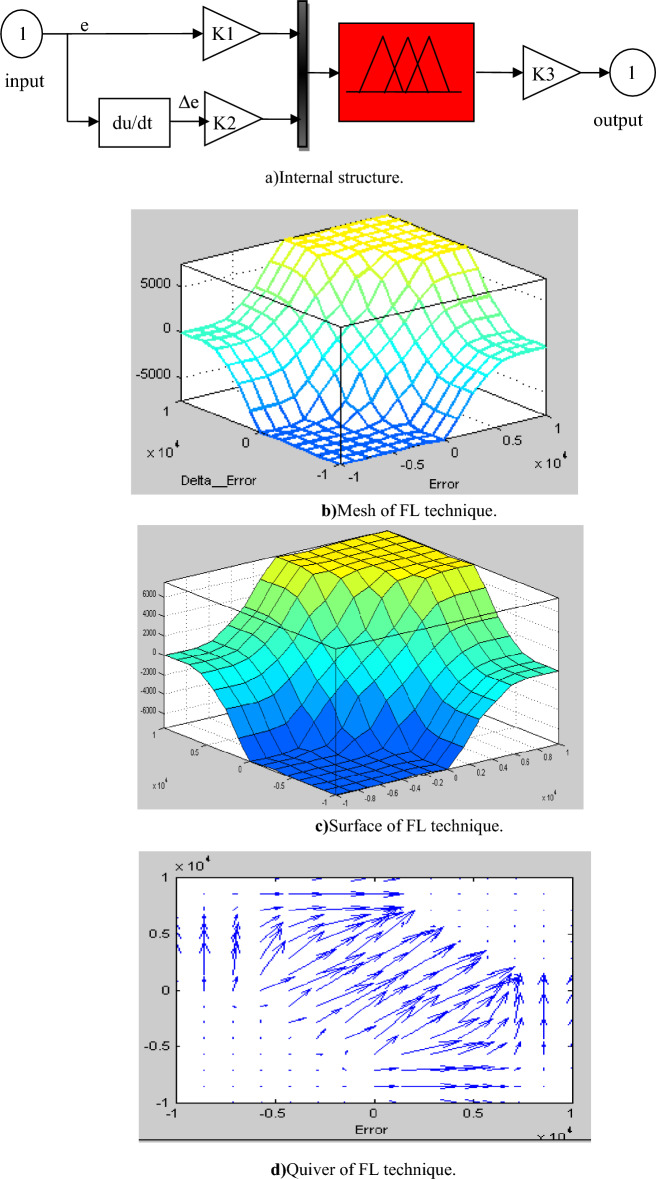
FL technique.

The internal structure of the FL controller is represented in Fig. 5, where there are three gains (K_1_, K_2_, and K_3_) used to adjust and change the proposed controller response. In Fig. 5 the surface, mesh and quiver of the used FL controller are also shown. The FL controller used has two inputs and one output. FL controller was chosen as a suitable solution because of its robustness, ease of application, outstanding performance, and unaffected by parameter changes. The method of experimentation and simulation was used to determine the gain values of the FL controllers and the gains that provided the best results in terms of power quality and current were taken.

**Figure 6:**
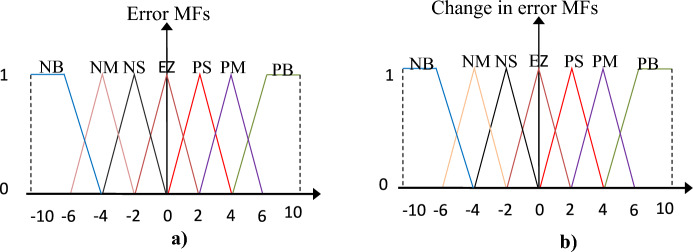
MFs of inputs.

All the FL controllers used have the same structure represented in Fig. 5a, using the same number of rules.49 rules were used to implement the FL controls used in the designed technique, as Table 4 shows these rules. This number of rules was chosen to obtain a fast dynamic response and obtain the greatest efficiency and performance to reduce power ripples and the value of current THD. Figure 6 represents the seven FL controller membership functions (MFs) used for input variables (error and change in error)^55,56^.

**Table 4:** FL controller rules^55^.

e	NB	NM	NS	EZ	PS	PM	PB
∆e							
NM	NB	NB	NB	NM	NS	EZ	PS
EZ	NB	NM	NS	EZ	PS	PM	PB
PB	EZ	PS	PM	PB	PB	PB	PB
NB	NB	NB	NB	NB	NM	NS	EZ
PM	NS	EZ	PS	PM	PB	PB	PB
NS	NB	NB	NM	NS	EZ	PS	PM
PS	NM	NS	EZ	PS	PM	PB	PB

In Table 5, the characteristics of the FL strategy used to achieve the proposed control are summarized. These characteristics can be changed depending on the system used for the study.

**Table 5:** Parameters of the FL controller.

Implication	Min
FIS type	Mamdani
And technique	Min
Or technique	Max
Defuzzification	Centroid
Aggregation	Max

## Results and discussions

In this section, the CFPC-PWM technique will be implemented using MATLAB software (https://www.mathworks.com/products/new_products/release2022a.html), with results compared to the DPC-PI technique in terms of THD of current, response time, energy undulations minimization ratio, overshoot, and SSE. Therefore, three tests are proposed to study the comparison between the CFPC-PWM technique and DPC-PI. Also, two different WS profiles are used to study the efficiency, effectiveness, and performance of the proposed CFPC-PWM strategy, where the simulation time for the first and second tests was 0.63 s and for the third test was 2.2 s.To accomplish this work, ode4 (Runge–kutta) solver was used. Also, the type solver option is Fixed-step. For both controls, Fixed-step size: 1e-5 was used. Also, unconstrained and auto were used for Periodic sample time constraints and tasking mode for periodic sample times, respectively. This section ends with a comparative study between the completed work and some existing work. The values of the DFIG parameters used in the simulation are given in Table 6^1,26^.

**Table 6:** DFIG parameters.

Parameter	Values
*R*_*s*_	12 mΩ
*P*_*sn*_	1500 kW
*L*_*r*_	13.6 mH
*L*_*m*_	13.5 mH
p	2
*J*	1 Mg m^2^
*L*_*s*_	13.7 mH
*R*_*r*_	21 mΩ
*f*_*r*_	2.4 mN m/s
*Vs*	380/696 V
*fs*	50 Hz

### First test case

**Figure 7:**
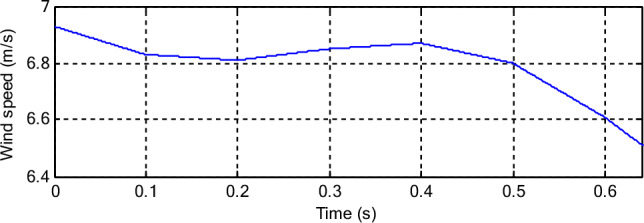
WS profile (Test 1).

**Figure 8:**
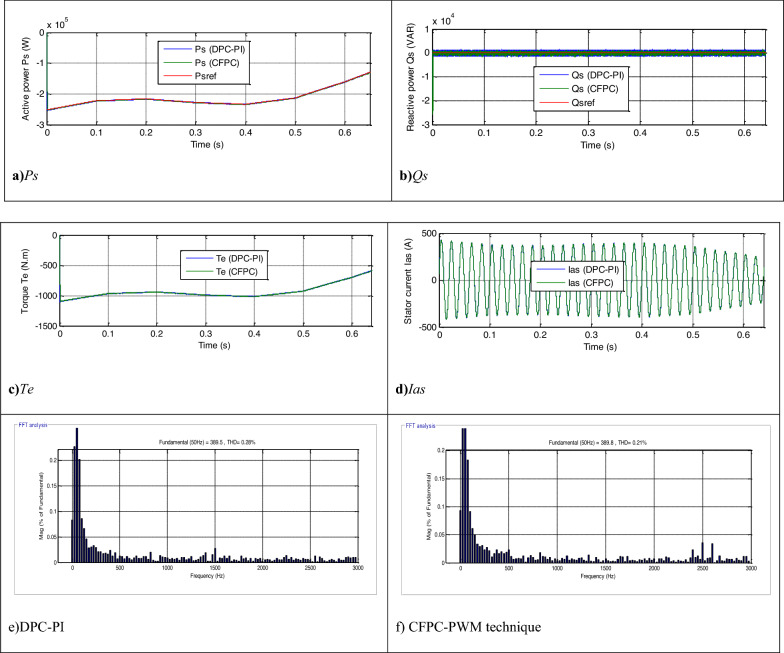
Results in the first test.

The CFPC-PWM technique is tested in the case of a variable WS, where the WS is used according to Fig. 7, and the characteristics of both techniques are studied in terms of tracking references. The necessary graphical and numerical results are extracted for this purpose. The results obtained are represented in Fig. 8, where it is noted that the energies follow the references well (Fig. 8a,b). Also, the *Ps* changes according to the change in WS, but the *Qs* is not affected by the change in WS and remain constant throughout the simulation period. In addition, ripples are observed in the case of both techniques.

A Fig. 8c,d represent torque and current, respectively. Through these two forms, torque and current change according to the change in WS, as their value increases with increasing WS and decreases with decreasing WS.The current takes a sinusoidal shape for both techniques. The THD value of the current for both techniques is represented in two Fig. 8e,f, where the THD for the CFPC-PWM technique was 0.21% and for the DPC-PI was 0.28%. Accordingly, the CFPC-PWM technique significantly minimized the THD by 25%, resulting in improved current quality compared to the DPC-PI technique. Also, it is noted that the two controls have almost the same amplitude value of the fundamental (50 Hz) of current signal with an advantage for the proposed CFPC-PWM strategy, as the amplitude value was 389.5 A and 389.8 A for both the DPC-PI technique and CFPC-PWM strategy, respectively.

**Figure 9:**
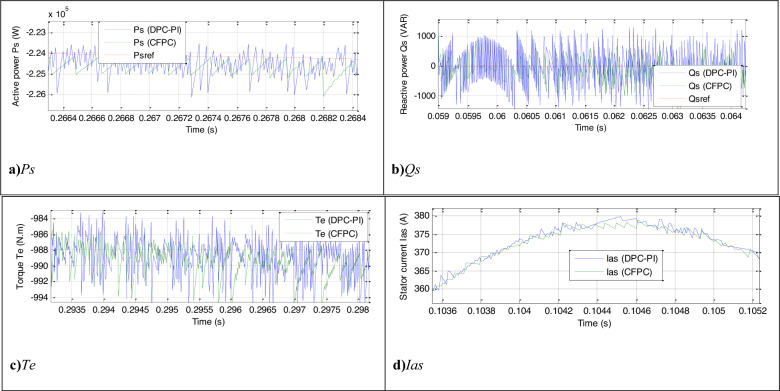
Zoom in the results of the first test.

Figure 9 represents a zoom of the results of the first test, where it is noted that the power, current, and torque ripples are larger in the case of the DPC-PI compared to the CFPC-PWM technique. The power ripples, response time, SSE, and DFIG overshoot are listed in Table 7. The CFPC-PWM strategy reduced the SSE value of DFIG power compared to the DPC-PI strategy by ratios estimated at 85.71% and 86.60% for both *Ps* and *Qs*, respectively. Also, the power ripples were improved compared to the DPC-PI strategy by 37.50% and 39.02% for both *Ps* and *Qs*, respectively. In the case of overshoot, the proposed CFPC-PWM strategy provided satisfactory results for *Ps*(65.56%) and unsatisfactory results for *Qs* (−72.96%) compared to the DPC-PI strategy. The CFPC-PWM strategy provided a better response time for *Qs* than the DPC-PI strategy, as this time was reduced compared to the DPC-PI strategy by an estimated ratio of 83.76%. However, in the case of *Ps*, the CFPC-PWM strategy provided an unsatisfactory time compared to the DPC-PI strategy. The latter reduced the *Ps* response time by an estimated 38.76% compared to the proposed CFPC-PWM strategy. This negativity of the proposed CFPC-PWM strategy can be attributed to the values of control gains, where the DPC-PI method was used to determine them.

**Table 7:** Numerical results in the first test.

	Techniques	*Ps* (W)	*Qs* (VAR)
SSE	DPC-PI	840	630.24
	CFPC-PWM technique	120	84.45
	Ratios (%)	85.71	86.60
Ripples	DPC-PI	1600	2624
	CFPC-PWM technique	1000	1600
	Ratios(%)	37.50	39.02
Overshoot	DPC-PI	2410	140.25
	CFPC-PWM technique	830	518.74
	Ratios (%)	65.56	−72.96
Response time (ms)	DPC-PI	1.09	1.078
	CFPC-PWM technique	1.78	0.175
	Ratios (%)	−38.76	83.76

### Second test case

**Figure 10:**
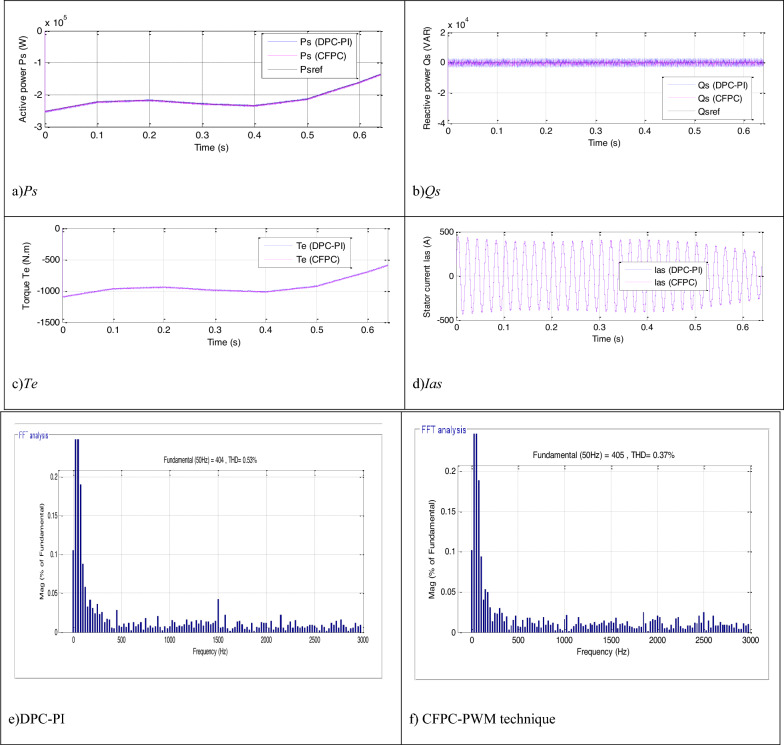
Results in the second test.

In this test, the same WS change form used in the first test is used.The robustness of the CFPC-PWM technique is studied in this test, where the DFIG parameters are changed according to Table 8. This test aims to determine the robustness of the proposed CFPC-PWM strategy compared to the DPC-PI strategy. The graphical results are shown in Fig. 10. The powers continue to follow the references despite the change in the DFIG parameters for the two techniques (Fig. 10a,b), with an increase in ripples and the current THD being observed. Torque and current have the form of *Ps*and their value is related to the change in WS despite the change in the DFIG parameters (Fig. 10c,d). Also, the current remains sinusoidal for the two strategies. In Fig. 10e,f, it is noted that the THD value was 0.53% and 0.37% for the DPC-PI and CFPC-PWM techniques, respectively. So, the CFPC-PWM technique minimized THD by an estimated 30.18% compared to the DPC-PI technique. On the other hand, it is noted that the proposed CFPC-PWM strategy gave a larger amplitude for the fundamental signal (50 Hz) of current compared to the DPC-PI strategy, as the amplitude value was 404 A and 405 A for both the DPC-PI and proposed CFPC-PWM strategy, respectively. So, according to these values, the proposed CFPC-PWM strategy can improve the current quality despite changing the DFIG-MRWT parameters, which is a positive thing.

**Table 8:** New values for the DFIG parameters.

	*Ls*	*Lm*	*Rs*	*Rr*	*Lr*
Old values	13.7 mH	13.5 mH	12 mΩ	21m Ω	13.6 mH
New values	6.85 mH	6.75 mH	24 mΩ	42 mΩ	6.8 mH

**Figure 11:**
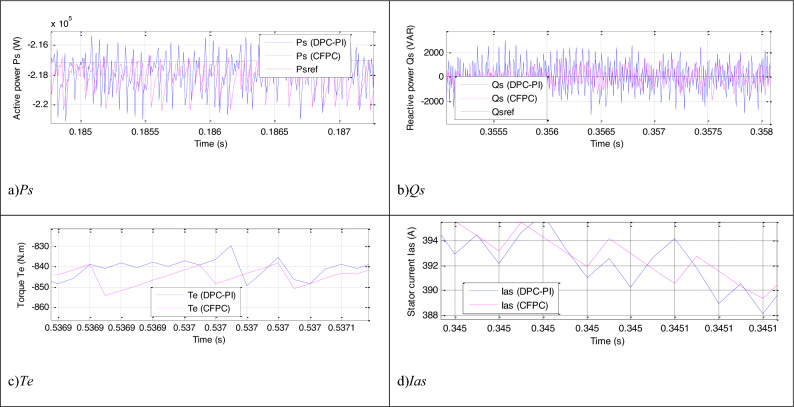
Zoom in the results of the second test.

The energy ripples, torque, and current of DFIG-MRWT are represented in Fig. 11. It is noted that these ripples are large in the case of the DPC-PI technique compared to the CFPC-PWM technique. This showed that the CFPC-PWM technique has better performance in enhancing the features of the energy system. The numerical values of the energy ripples are listed in Table 9, where the necessary reduction ratios were calculated to show how much the proposed CFPC-PWM strategy can reduce these ripples compared to the DPC-PI strategy. Also, the values and ratios of reduction for response time, overshoot, and SSE of DFIG power are given in this table. When examining the table, it was noted that the CFPC-PWM technique reduced the size of the power ripples by ratios estimated at 32.20% and 41.66% for both *Ps* and *Qs*, respectively, compared to the DPC-PI strategy. Also, the SSE of DFIG power was reduced by ratios estimated at 83.67% and 57.33% for both *Ps* and *Qs*, respectively, compared to the DPC-PI strategy. The latter gave better results than the CFPC-PWM technique in terms of *Ps* response time, as this time was reduced by an estimated 40.42% compared to the proposed CFPC-PWM strategy. However, the proposed CFPC-PWM strategy gave a better *Qs* time than the DPC-PI strategy, as this reduction was estimated at a ratio of 65.02% compared to the DPC-PI strategy. In terms of overshoot of DFIG power, the proposed CFPC-PWM strategy gave a better value for overshoot of *Ps* compared to the DPC-PI technique, as the reduction ratio was estimated at 41.39%, which is a good ratio. But the proposed CFPC-PWM strategy gave an unsatisfactory value for the overshoot of *Qs* compared to the DPC-PI technique, as the DPC-PI strategy reduced this value by an estimated ratio of 73.39% compared to the proposed CFPC-PWM strategy, which is a negative matter that can be attributed to the gains. This negativity can be overcome in the future by using smart strategies in determining gain values.

**Table 9:** Numerical results of the second test case.

	Techniques	*Ps* (W)	*Qs* (VAR)
SSE	DPC-PI	1470	3000
	CFPC-PWM technique	240	1280
	Ratios (%)	83.67	57.33
Ripples	DPC-PI	5000	6000
	CFPC-PWM technique	3390	3500
	Ratios (%)	32.20	41.66
Overshoot	DPC-PI	2730	83.15
	CFPC-PWM technique	1600	319.80
	Ratios	41.39	−73.99
Response time (ms)	DPC-PI	0.56	0.509
	CFPC-PWM technique	0.94	0.178
	Ratios	−40.42	65.02

In Table 10, a comparative study is completed between the two controls in terms of the degree to which the values of THD of current and amplitude of fundamental (50 Hz) are affected. From Table 10, it is noted that the value of THD has changed in the second test compared to the first test, as its value is noted to have increased in the case of the two controls. In the CFPC-PWM strategy, it increased by 0.16, and in DPC-PI technique, it increased by 0.25. Therefore, the CFPC-PWM strategy is better than DPC-PI technique in terms of changing the THD value, as the ratio change in the THD value was 47.16% and 43.24% for both the DPC-PI strategy and CFPC-PWM technique, respectively. Therefore, CFPC-PWM technique has higher durability in terms of improving current quality. On the other hand, the amplitude of the fundamental (50 HZ) signal changed its value in the second test for the two controls (Table 10), where an increase in the amplitude value was observed. This increase was estimated at 3.75% and 3.58% for both the proposed CFPC-PWM and DPC-PI strategies, respectively. Therefore, the proposed CFPC-PWM strategy provided a greater ratio, which indicates that the amplitude changed more if the proposed CFPC-PWM strategy was used compared to the DPC-PI strategy.

**Table 10:** Study of the change in the THD and amplitude value of the fundamental (50 Hz) for the both techniques.

		Techniques	
		DPC-PI strategy	CFPC-PWM technique
THD (%)	Test 1	0.28	0.21
	Test 2	0.53	0.37
	Test 2—Test 1	0.25	0.16
	Ratios	47.16%	43.24%
Amplitude value of the fundamental (50 Hz)	Test 1	389.50 A	389.80 A
	Test 2	404 A	405 A
	Test 2—Test 1	14.50 A	15.20 A
	Ratios	3.58%	3.75%

In Table 11, the ratios of change in the values of ripples, response time,overshoot, and SSE of DFIG energy in the two tests relative to the two controls together are given. These ratios are calculated according to Eqs. (25) to (28). This table gives a clear picture of the change in the values of ripple, response time, SSE, and overshoot for the two techniques used, where the ratios of change in these values between the first and second tests are calculated. These ratios demonstrate the extent to which the CFPC-PWM strategy is affected compared to the DPC-PI strategy in terms of changing system parameters. The CFPC-PWM strategy provided greater change in SSE of DFIG power compared to DPC-PI. Also, the proposed CFPC-PWM strategy provided a lower ratio of change in the *Qs* ripples than the DPC-PI strategy, as this ratio was estimated at 56.26% and 54.28% for both the DPC-PI and proposed CFPC-PWM strategies, respectively. However, the proposed CFPC-PWM strategy provided a greater ratio of change in the *Ps* ripples than the DPC-PI strategy, as this ratio was estimated at 68% and 70.50% for both the DPC-PI and proposed CFPC-PWM strategies, respectively.In the case of overshoot of DFIG power, it is noted that in the two controls, the value of overshoot of *Qs* decreased in the second test compared to the first test, as this decrease was estimated at a rate of 40.71% and 38.35% for both the DPC-PI and the proposed CFPC-PWM strategy, respectively. Therefore, the proposed CFPC-PWM strategy provided the lowest ratio. But in terms of the overshoot value of *Ps*, it is noted that this value increased in the second test compared to the first test for the two controls, where this increase was estimated at ratios of 11.72% and 48.12% for both the DPC-PI and proposed CFPC-PWM strategy, respectively. Therefore, the proposed CFPC-PWM strategy provided the largest ratio compared to the DPC-PI strategy. The response time values for the power in the case of DPC-PI control were noted to have decreased in the second test compared to the first test due to the change in the DFIG parameters. This decrease was estimated at ratios of 52.78% and 48.62% for both *Ps* and *Qs*, respectively. In the case of the proposed CFPC-PWM strategy, it is noted that the response time of the *Ps*decreased in value in the second test compared to the first test, as this decrease was estimated at 47.19%. Therefore, the proposed CFPC-PWM strategy provided a lower declineratio than the DPC-PI strategy. However, the response time of the *Qs* increased slightly in the second test compared to the first test, where this increase was estimated at 1.68%. According to these ratios presented, it can be said that the proposed CFPC-PWM strategy provided better impact rates and unsatisfactory impact rates compared to the DPC-PI strategy, which is normal, as each control strategy has negatives and positives.

25 $$A\left(\%\right)=\frac{{X}_{{s}_{ripple}}(test 2)-{X}_{{s}_{ripple}}(test 1)}{{X}_{{s}_{ripple}}(test 2)}$$

26 $$B\left(\%\right)=\frac{{X}_{{s}_{overshoot}}(test 2)-{X}_{{s}_{overshoot}}(test 1)}{{X}_{{s}_{overshoot}}(test 2)}$$

27 $$C\left(\%\right)=\frac{{X}_{{s}_{SSE}}(test 2)-{X}_{{s}_{SSE}}(test 1)}{{P}_{{s}_{SSE}}(test 2)}$$

28 $$D\left(\%\right)=\frac{{X}_{{s}_{responsetime}}(test 2)-{X}_{{s}_{responsetime}}(test 1)}{{X}_{{s}_{responsetime}}(test 2)}$$

where, X is a quantity that can be *Ps* or *Qs*.

**Table 11:** Robustness analysis.

Robustness indicator	Analyzed control techniques			
	DPC-PI		CFPC-PWM	
	*Qs* (VAR)	*Ps* (W)	*Qs* (VAR)	*Ps* (W)
A (%)	56.26	68	54.28	70.50
B (%)	−40.71	11.72	−38.35	48.12
C (%)	78.99	42.85	93.40	50
D (%)	−48.62	−52.78	1.68	−47.19

### Third test case

**Figure 12:**
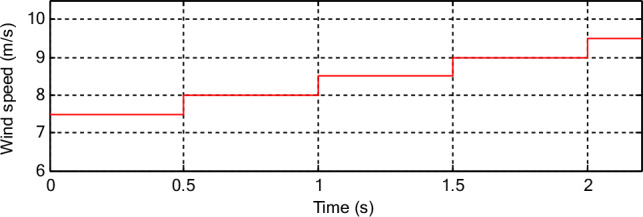
Steps WS profile.

**Figure 13:**
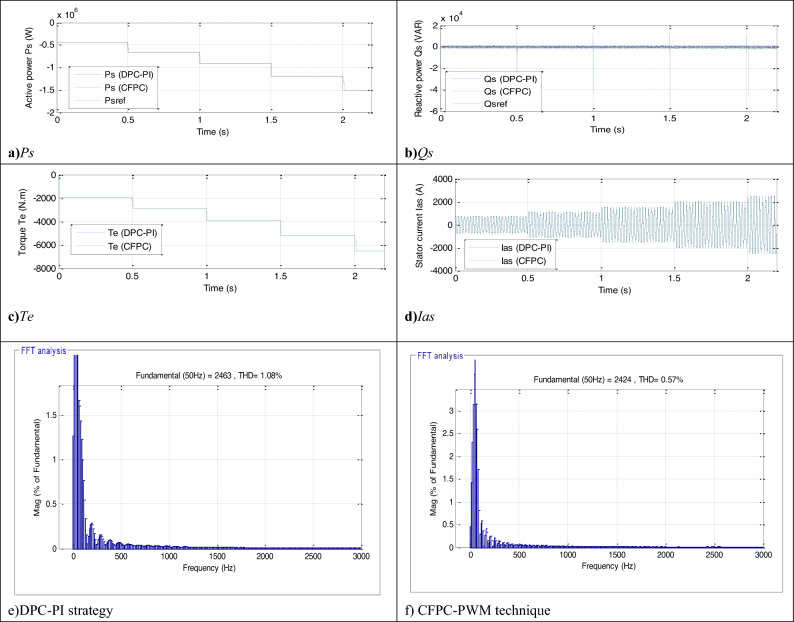
Results in the third test.

**Figure 14:**
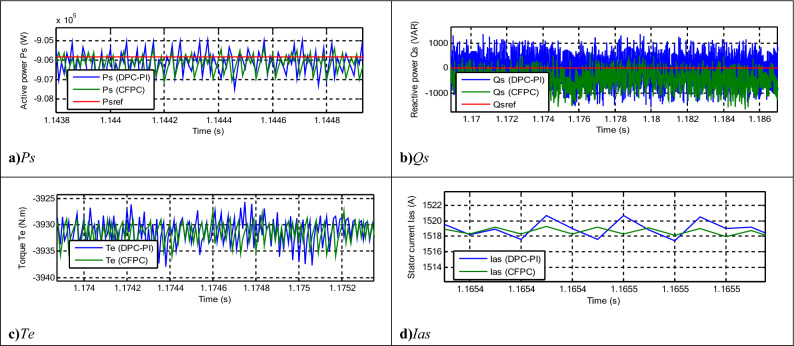
Zoom in the results of the third test.

This test differs from the two tests above in terms of the form of WS change, as a form of WS change different from the form of WS change used in the first test is used. In Fig. 12, the WS profile used in this test is listed, where the WS variation profile is in steps. In Figs. 13 and 14 the graphical results are listed and the numerical results are listed in Table 12. According to Fig. 13, the capacities continue to follow the references well and are the same as the results of the previous tests, with the presence of ripples. The *Ps* of the two controllers changes according to the change in WS, and the *Qs* does not change according to the change in WS, as its value remains constant and equal to 0 VAR. The value of torque and current changes according to the change in the shape of the WS, as it decreases and increases as the WS decreases and increases with the presence of ripples in both the two controls. Also, the current has a sinusoidal shape in the case of the two controls, which is the same as the results of the previous two tests.

**Table 12:** Numerical results in the third test.

	Techniques	*Ps* (W)	*Qs* (VAR)
SSE	DPC-PI	900	1000
	CFPC-PWM technique	400	277.40
	Ratios (%)	55.55	72.26
Ripples	DPC-PI	2600	3000
	CFPC-PWM technique	1600	2054.65
	Ratios (%)	38.46	31.51
Overshoot	DPC-PI	3050	388.52
	CFPC-PWM technique	2980	518.60
	Ratios (%)	2.29	-25.08
Response time (ms)	DPC-PI	2.03	2.04
	CFPC-PWM technique	4.07	0.175
	Ratios (%)	−50.12	91.42

The current THD for the two controls is listed in Fig. 13e,f. This value was 0.57% and 1.08% for both the proposed CFPC-PWM and DPC-PI strategies, respectively. So, the proposed CFPC-PWM strategy reduced the THD value compared to the DPC-PI strategy by an estimated rate of 47.22%, which is a high ratio and indicates that the quality of the current is better in this test if the proposed CFPC-PWM strategy is used compared to the DPC-PI strategy. Also, it is noted that the amplitude value of the fundamental signal (50 Hz) was 2424 A and 2463 A for both the proposed CFPC-PWM and DPC-PI strategies, respectively. So, the proposed CFPC-PWM strategy presented a lower duration in this test than the DPC-PI strategy, which is undesirable.

In Fig. 14, the ripples of power, torque, and current for the two controls are shown. Therefore, the ripples are low when using the proposed CFPC-PWM strategy compared to the DPC-PI strategy, as the values of these ripples are listed in Table 12. This table gives the reductions for response time, ripple, SSE, and DFIG overshoot. From this table, it is noted that the CFPC-PWM technique provided good results in terms of ripples and SSE of powers and this is shown by the calculated ratios. The CFPC-PWM strategy reduced power ripples compared to the DPC-PI strategy by ratios estimated at 31.51% and 38.46% for both *Qs* and *Ps*, respectively. Also, the SSE value of the compared powers was reduced compared to the DPC-PI strategy by ratios estimated at 72.26% and 55.55% for both *Qs* and *Ps*, respectively. On the other hand, the CFPC-PWM strategy reduced the response time of *Qs* and overshoot of active power compared to the DPC-PI strategy by ratios estimated at 91.42% and 2.29%, respectively. These ratios show the superiority of the proposed CFPC-PWM strategy in improving the characteristics of the studied energy system. However, this strategy provided unsatisfactory results in terms of response time to *Ps* and overshoot of *Ps*, which is a negative matter that can be overcome in the future by using smart strategies such as NNs.

In Table 13, the change in the values of both current THD and amplitude of fundamental (50 Hz) is studied, as it is noted that these two values changed in the third test compared to the first test. So, these two values are affected by the change in the shape of the WS, as it is noted that these two values increased significantly in the third test. In the case of the THD value, this increase was estimated at 70.07% and 63.15% for both the DPC-PI and the proposed CFPC-PWM strategy, respectively. Accordingly, the proposed CFPC-PWM strategy presented a lower ratio, which indicates that it is less affected than the DPC-PI strategy, which is a positive thing that indicates its superiority. In terms of the value of the amplitude, both strategies presented a significant increase in the value of this amplitude in the third test compared to the first test. These increases were estimated at 84.18% and 83.91% for both the DPC-PI and the proposed CFPC-PWM strategy, respectively. So the CFPC-PWM provided a lower ratio than the DPC-PI strategy, which indicates that this strategy is more robust and efficient than the DPC-PI strategy and, therefore, can be relied upon in the future in the field of control.

**Table 13:** Study of the change in the amplitude value of the fundamental (50 Hz) and THD for both techniques (first and third tests).

		Strategies	
		DPC-PI strategy	CFPC-PWM technique
THD (%)	Test 1	0.28	0.21
	Test 3	1.08	0.57
	Test 3—Test 1	0.80	0.36
	Ratios	74.07%	63.15%
Amplitude value of the fundamental (A)	Test 1	389.50	389.80
	Test 3	2463	2424
	Test 3—Test 1	2073.49	2034.20
	Ratios	84.18%	83.91%

Finally, this proposed work is concluded with a comparative study of existing works related to DFIG. This analysis is highly significant as it provides a different perspective on the CFPC-PWM technique and its effectiveness in enhancing the features of DFIG in comparison to other studies. A comparison is made between other works in terms of ripple reduction ratios, overshoot, and SSE of DFIG power. Also, by comparing the response time of the powers, SSE, and current THD. The comparison results are listed in Tables 14, 15, 16 and 17. Through these completed tables, it is noted that the CFPC-PWM technique provided better reduction rates than several scientific works, which proves its distinguished performance. These tables give a clear picture of the superiority of the CFPC-PWM technique and its great ability to improve energy quality compared to other strategies. So, this strategy can be relied upon in the future in the field of command.

**Table 14:** Comparison in terms of response time for DFIG power.

References		Time response (ms)	
		*Ps*	*Qs*
^58^		15	80
^59^		–	28
^60^	DPC	17	18
	Nonlinear DPC technique	9	5
^61^		32	–
^48^		33.8	34.5
CFPC-PWMtechnique	Test 1	1.78	0.175
	Test 2	0.94	0.178
	Test 3	4.07	0.175

**Table 15:** Comparison in terms of power ripple minimization rates.

References		Ratios(%)	
		*Qs*	*Ps*
^51^		36.93	22.95
^62^		35	36
^63^	STC	22.66	21.75
	Modified STC	21.23	19.11
^64^		46.93	28.57
CFPC-PWM technique	Test 1	39.20	37.50
	Test 2	41.66	32.20
	Test 3	31.51	38.46

**Table 16:** Comparison in terms of current THD.

References		THD (%)
^65^		3
^66^		4.2
		4.9
^67^		3.1
^68^		1.66
^69^		3.7
^70^		3.13
^71^		10.79
		4.05
^72^		9.7
		3.2
CFPC-PWM technique	Test 1	0.21
	Test 2	0.37
	Test 3	0.57

**Table 17:** Comparison in terms of SSE for DFIG energy.

References		SSE ratios (%) of DFIG energy	
		*Qs*	*Ps*
^45^		80	77.27
^62^		35.48	62
^51^		36.93	35
^52^		42.14	47.57
^73^	Test 1	78.44	45.83
	Test 2	52.22	56.52
	Test 3	48.75	87.50
^74^	Test 1	53.25	74.41
	Test 2	52.98	79.55
	Test 3	45.74	94.81
^75^	Test 1	46.86	63.96
	Test 2	45.48	78
	Test 3	43.21	60.03
^76^	Test 1	41.66	52.28
	Test 2	45.83	61.25
	Test 3	55.17	44.18
CFPC-PWMtechnique	Test 1	86.60	85.71
	Test 2	57.33	83.67
	Test 3	72.26	55.55

## Conclusions

A new control based on CFPC strategy and PWM technique was introduced to control the DFIG power and improve the characteristics of the energy system based on a MRWT. The proposed CFPC-PWM technique was compared with the DPC-PI technique and other existing techniques, where MATLAB software (https://www.mathworks.com/products/new_products/release2022a.html) was used to implement this suggested technique using different WS profiles. The behavior of the CFPC-PWM technique was studied in terms of reference tracking, current THD, robustness, ripple reduction rates, response time, overshoot, and SSE of DFIG power. The results obtained from this work can be summarized in the following points:

- Reducing the THD value of the current compared to the DPC-PI technique, as the reduction ratio in thesuggested tests was estimated at 25%, 30.18%, and 47.22%;
- The proposed CFPC-PWM strategy is considered a new, more efficient and reliable strategy in the field of power control than the DPC-PI strategy;
- Significantly increasing the robustness of the DFIG-MRWT system.
- Reducing the value of *Ps* ripples compared to DPC-PI, as the ratio of reduction in the proposed tests was estimated at 37.50%, 32.20%, and 38.46%;
- Minimizing the both SSE and overshoot valuesofDFIG energy.

In the future, other new strategies based on the combination of different controls will be implemented to control MRWT systems. Also, in addition to this work, the strategy of the cascaded NN technique will be experimentally implemented to compare the simulation results with those obtained experimentally, including in other works.

## Data Availability

Data available on request from the authors. The datasets used and/or analysed during the current study available from the corresponding author on reasonable request. In the event of communication, the first author (Habib Benbouhenni, E-mail: habib.benbouenni@nisantasi.edu.tr) will respond to any inquiry or request.

## Abbreviations

PI: Proportional-integral controller
NN: Neural network
FL: Fuzzy logic
WT: Wind turbine
WS: Wind speed
EE: Electrical energy
*Ps*: Active power
PV: Photovoltaic system
ST: Switching table
HC: Hysteresis controller
RSC: Rotor side converter
SVM: Space vector modulation
GA: Genetic algorithm
SSE: Steady-state error
MRWT: Multi-rotor wind turbine
DFIG: Doubly-fed induction generator
THD: Total harmonic distortion
*Qs*: Reactive power
RESs: Renewable energy sources
WE: Wind energy
SMC: Sliding mode control
DPC: Direct power control
GSC: Grid side converter
CFPC: Cascaded fuzzy power control
BC: Backstepping control
PWM: Pulse width modulation
STC: Super-twisting control
MPPT: Maximum power point tracking
